# A hierarchical spatiotemporal analog forecasting model for count data

**DOI:** 10.1002/ece3.3621

**Published:** 2017-12-07

**Authors:** Patrick L. McDermott, Christopher K. Wikle, Joshua Millspaugh

**Affiliations:** ^1^ Department of Statistics University of Missouri Columbia MO USA; ^2^ Wildlife Biology Program University of Montana Missoula MT USA

**Keywords:** ecological forecasting, hierarchical Bayesian models, nonlinear forecasting, waterfowl settling patterns

## Abstract

Analog forecasting is a mechanism‐free nonlinear method that forecasts a system forward in time by examining how past states deemed similar to the current state moved forward. Previous applications of analog forecasting has been successful at producing robust forecasts for a variety of ecological and physical processes, but it has typically been presented in an empirical or heuristic procedure, rather than as a formal statistical model. The methodology presented here extends the model‐based analog method of McDermott and Wikle (Environmetrics, 27, 2016, 70) by placing analog forecasting within a fully hierarchical statistical framework that can accommodate count observations. Using a Bayesian approach, the hierarchical analog model is able to quantify rigorously the uncertainty associated with forecasts. Forecasting waterfowl settling patterns in the northwestern United States and Canada is conducted by applying the hierarchical analog model to a breeding population survey dataset. Sea surface temperature (SST) in the Pacific Ocean is used to help identify potential analogs for the waterfowl settling patterns.

## 1 INTRODUCTION

Contemporary issues in natural resource management such as climate change rely increasingly on quantitative forecasts at time scales ranging from seasonal to decadal (e.g., LeBrun, Thogmartin, Thompson, Dijak, & Millspaugh, 2016). There are great challenges when making such forecasts in a rapidly changing environment. One of the most important challenges to policy and management is to quantify the uncertainty of the forecasts (e.g., Clark et al., 2001; Conroy, Runge, Nichols, Stodola, & Cooper, 2011; and references therein). There are many potential issues with quantifying uncertainty, related to the characterization of uncertainties in data, mechanistic processes, and interactions across biological and physical systems (e.g., Oliver & Roy, 2015). Perhaps surprisingly, in many cases, the best forecast models rely on nonparametric and “mechanism‐free’’ specifications (e.g., Perretti, Munch, & Sugihara, 2013; Ward, Holmes, Thorson, & Collen, 2014). Bayesian models in general, and Bayesian hierarchical models in particular, provide a comprehensive modeling framework which account for multiple sources of uncertainty in ecological models (e.g., Wikle, 2003; Royle & Dorazio, 2008; Cressie, Calder, Clark, Hoef, & Wikle, 2009; to name a few); for a historical overview, see Ellison (2004). To date, there have been few attempts to cast “mechanism‐free’’ models within the Bayesian framework (McDermott & Wikle, 2016).

Quantifying uncertainty for spatial–temporal ecological processes is complicated because the evolution of these processes over time is often nonlinear. One mechanism‐free solution to the spatiotemporal forecasting problem is known as “analog forecasting’’ (e.g., Lorenz, 1969). Analog forecasting uses past states of a system that are similar to the current state and then assumes that the current state of the system will evolve in a manner similar to how the identified past states evolved. For our purposes, an analog refers to a past state of some system that is similar to the current state of the system. Analog forecasting is appealing for dynamical processes governed by some underlying, but unspecified, deterministic law. Specifically, analog forecasting leverages the predictability in these types of systems by finding past trajectories similar to the current trajectory of the system.

Much of the current development of spatiotemporal analog methods utilizes the idea of embedding a dynamical system in time, similar to the simplex prediction method outlined in Sugihara and May (1990) for univariate time series. Indeed, the Sugihara and May (1990) approach was one of the first practical methods to introduce the idea of embedding a dynamical system in the context of nonlinear forecasting. Their methods utilized the state‐space dynamical system reconstruction theory of Takens (1981). In complicated dynamical systems, one rarely observes all of the state variables. State‐space reconstruction allows one to reconstruct a dynamical system with only a subset of the state variables, by considering those state variables at multiple lags in the past. As dynamical systems evolve in time, they tend to revisit previous paths in the phase space, where these paths live on some low‐dimensional manifold of the entire space (i.e., the attractor). Thus, through the use of state‐space reconstruction, one can recover features of past dynamical paths along the attractor. Sugihara and May (1990) recognized the utility of state‐space reconstruction within the context of nonlinear forecasting. In particular, they showed how embedding vectors, created by lagging past states of a system (historical data) in time (e.g., see Chapter 3 of Cressie & Wikle, 2011), could be utilized to find robust analogs for the current state of the system. In an ecological context, Takens’ theorem (Takens, 1981) has also been utilized to analyze the relationships between multiple components of an ecological system in Nichols, Moniz, Nichols, Pecora, and Cooch (2005), and more recently in Sugihara et al. (2012). This remarkably simple forecasting method has proved successful in a multitude of time series applications (e.g., Perretti et al., 2013; Sugihara et al., 2012; Zhao & Giannakis, 2014).

Mechanism‐free and analog methods traditionally have relied on nonparametric and/or heuristic approaches that did not include a formal probabilistic error structure (although, see Tippett & DelSole, 2013; Lguensat, Tandeo, Ailliot, Pulido, & Fablet, 2016). Modern nonparametric analog methods require choice of the embedding dimension of the analogs, the number of past analogs to consider, and weights for those analogs. All of these choices can significantly impact the analog forecast. For example, the question of how many past analogs to use can be thought of as a *k*‐nearest neighbor problem, where the neighborhood consists of the analogs most similar to the current state of the system. Given the number of “neighboring” analogs, a kernel defined by a smoothing parameter is typically used to determine the weights (e.g., McDermott & Wikle, 2016; Zhao & Giannakis, 2014). However, previous analog forecasting implementations have employed either some heuristic method that does not explicitly account for uncertainty associated with the choice or a multidimensional cross‐validation search (e.g., Arora, Little, & McSharry, 2013), to choose these values. The Bayesian framework described in McDermott and Wikle (2016) allows for both the estimation and incorporation of model averaging over the various parameters in the analog model, thereby accounting for the uncertainty induced by their selection.

Once framed within the context of Bayesian modeling, analog forecasting can be placed within the rich class of models available in the space‐time hierarchical Bayesian framework (e.g., Cressie & Wikle, 2011; Wikle, 2015), which allows for robust quantification of uncertainty. We present here a hierarchical analog forecasting model that extends the model developed in McDermott and Wikle (2016) to include a formal non‐Gaussian data model – specifically, a Poisson model to accommodate count data. This is the first analog method that accounts explicitly for non‐Gaussian data within a statistical framework. The model is applied to the problem of producing one‐year‐ahead forecasts of waterfowl settling patterns given the state of the Pacific Ocean sea surface temperature (SST). Because spatiotemporal analog forecasting can quickly become prohibitive for high‐dimensional processes, we introduce an approach for spatiotemporal dimension reduction in count data known as nonnegative matrix factorization (Lee & Seung, 2001).

## 2 MATERIALS AND METHODS

### 2.1 Waterfowl and sea surface temperature data

Migratory waterfowl settling patterns, productivity, and survival have been shown to depend strongly on climate‐related habitat conditions (e.g., Feldman, Anderson, Howerter, & Murray, 2015; Hansen & McKnight, 1964; Herter, 2012). Settling patterns of waterfowl are of substantial importance to wildlife ecologists. From a management perspective, knowledge of settling patterns aids in establishing appropriate harvest regulations across management units and in determining the efficacy of habitat treatments designed to improve habitat for waterfowl (i.e., Lavretsky, Miller, Bahn, & Peters, 2014). Further, given the migratory nature of waterfowl and ongoing theoretical interests in understanding factors affecting the distribution and habitat selection of migratory species, predicting settling patterns has broad relevance. It is known that changes to habitat conditions can lead to more flexible settling patterns along a latitudinal gradient that can mitigate site philopatry, and possibly decrease productivity or recruitment (e.g., Becker, 2015; Johnson & Grier, 1988; Karanth, Nichols, Sauer, Hines, & Yackulic, 2014). Given the well‐known relationships between Pacific Ocean (particularly the tropical ocean) SSTs and North American climate conditions (e.g., Philander, 1990) and the potential for these conditions to affect waterfowl settling patterns (Sorenson, Goldberg, Root, & Anderson, 1998), it is reasonable to use Pacific Ocean SST as a proxy for future habitat conditions. In addition, the impact of Pacific SSTs is typically nonlinear (Hoerling, Kumar, & Zhong, 1997), suggesting nonlinear evolution models are appropriate. Although others (e.g., Wu, Holan, & Wikle, 2013) have successfully forecast Mallard duck (*Anas platyrhyncho*) settling patterns using a drought severity index, we provide a one‐year forecast given the Pacific SSTs through the previous May.

Since 1955, the U.S. Fisheries and Wildlife Service (USFWS) and Canadian Wildlife Service (CWS) have jointly conducted a Breeding Population Survey (BPS) in the northern United States and Canada. A purpose of these surveys was to provide data that can be used in developing waterfowl harvest regulations. In addition to estimating status and trends of waterfowl populations, these data also aid in understanding waterfowl distributions. The BPS is the most expansive survey of waterfowl distributions in North America covering about 3.4 million square kilometers of land each year. Often, the U.S. Fish and Wildlife Service discusses waterfowl distribution results as they relate to climate data (e.g., temperatures and timing of precipitation), but there is rarely formal consideration of how these factors influence settling patterns. Each spring (mid‐ to late May) crew consisting of one pilot and one observer flies transect lines and records counts of various waterfowl species. For selected areas, ground crews also record counts to develop visibility correction factors. Each 400 m wide transect is divided into a series of segments measuring 29 km in length. The analysis conducted here consists of the 1,067 locations between 96–115°W longitude and 43–54°N latitude from 1970 through 2014. The majority of survey locations north of 54° latitude have little temporal variability, with zero counts in most years and are not considered. Although the BPS survey records counts for several species, we focus on raw indicator pair counts (i.e., counts of paired ducks and lone drakes) for mallards. The raw indicated pair counts are publicly available through the FWS Division of Migratory Management (https://migbirdapps.fws.gov/).

Monthly SST from 1970 to 2014 was obtained from the publicly available National Oceanic and Atmospheric Administration (NOAA) Extended Reconstruction Sea Surface Temperature (ERSST) data (http://www.esrl.noaa.gov/psd/). A subset of 3,132 locations from the ERSST data, between 30.5°S–60.5°N latitude and 123.5–290.5°E longitude with a spatial resolution of 2 × 2°, form the SST data. We follow the common procedure from the climate science literature by creating anomalies through the subtraction of location specific monthly means calculated from a climatological average spanning the period 1970–1999 (e.g., Wilks, 2011).

### 2.2 Spatiotemporal variables

Let $Y_{t}\left(s_{i}\right)$ be a component of a dynamical system at time *t* with spatial locations $\left\{s_{i},\;i=1,\ldots ,\;n_{y}\right\}$. Suppose we have access to data from the system for time periods {t=1,…,T}. The set of data at all *n*_*y*_ locations for time period *t* is defined as, $Y_{t}\equiv {\left(Y_{t}\left(s_{1}\right),\ldots ,\;Y_{t}\left(s_{n_{y}}\right)\right)}^{\prime }$. Here, we consider count‐valued data for $Y_{t}$. Further, we consider the use of some spatiotemporal forcing (predictor) variable, defined as, $x_{t^{\prime }}={\left(x_{t^{\prime }}\left(r_{1}\right),\ldots ,\;x_{t^{\prime }}\left(r_{n_{x}}\right)\right)}^{\prime }$, for spatial locations, $\left\{r_{1},\ldots ,\;r_{n_{x}}\right\}$ and time *t*′, to help forecast the process of interest (i.e., **Y**_*t*_). Note that the time indices *t* and *t*′ are separated by τ period(s) (i.e., τ=0,1,2,…), with potentially different time scales. As discussed in more detail below, in our application, τ represents the number of periods the response variable is forecasted into the future. Thus, the goal here is to forecast the value of $Y_{T+\tau }$ given values of $Y_{t}$ for t≤T and for $x_{t^{\prime }}$ for $t^{\prime }\leq T$. This is performed by weighting the past values of **Y**_*t*_ based on how well corresponding past sequences of $x_{t^{\prime }}$ match the most recent sequence of $x_{t^{\prime }}$ (i.e., the most recent sequence up to time *T*), as described below.

Many spatiotemporal dynamical processes can be challenging to model due to the high‐dimensional nature of the spatial component. Both the BPS waterfowl settling pattern data and SST data described above can be considered high dimensional. To efficiently model such spatiotemporal processes, some form of dimension reduction is usually performed (e.g., see Chapter 7 of Cressie & Wikle, 2011). Common methods such as empirical orthogonal functions (EOFs) are not ideal for noncontinuous responses such as count data because it is difficult to impose constraints (e.g., such as nonnegativity). Although more general ordination methods such as principal coordinate analysis and multidimensional scaling can be useful for noncontinuous data (e.g., Ellison & Gotelli, 2004), these methods also do not guarantee, in general, that after dimension reduction and projection back into physical space, the resulting process has the same support as the original data.

### 2.3 Response vector dimension reduction

Consider the case where we have *n*_*y*_ spatial locations and the *n*_*y*_‐dimensional response vector at time *t*,** Y**_*t*_. We seek a $n_{\beta }$‐dimensional expansion coefficient vector, $\beta_{t}$, associated with a set of $n_{\beta }$ basis functions $\left\{\psi_{j},\;j=1,\ldots ,\;n_{\beta }\right\}$, where $\psi_{j}\equiv {\left(\psi_{j}\left(s_{1}\right),\ldots ,\;\psi_{j}\left(s_{n_{y}}\right)\right)}^{\prime }$. In particular, we seek a reduced dimension representation such that $n_{\beta }<<n_{y}$. When considering a linear basis expansion, then, we seek $Y_{t}\;\approx \;\Psi \beta_{t}$, where $\Psi \equiv [\psi_{1},\ldots ,\;\psi_{n_{\beta }}]$ is a $n_{y}\;\times \;n_{\beta }$ matrix. Then, the ordinary least squares estimate of the expansion coefficients is ${\tilde{\beta }}_{t}={\left(\Psi^{\prime }\Psi \right)}^{-1}\Psi^{\prime }Y_{t}$, assuming $\left(\Psi^{\prime }\Psi \right)$ is invertible. In situations where Ψ is orthogonal, this simplifies to ${\tilde{\beta }}_{t}=\Psi^{\prime }Y_{t}$. As an example, Ψ derived from the scaled left singular vectors of a full data matrix, $Y\equiv [Y_{1},\ldots ,\;Y_{T}]$, are the EOF basis functions, and are orthogonal. A reduced rank representation of the response vectors in phase space is given by ${\tilde{Y}}_{t}=\Psi {\tilde{\beta }}_{t}$. Typically, one then considers the expansion coefficients, ${\tilde{\beta }}_{t}$, as the time‐varying variable of interest.

When $Y_{t}$ has a constrained support, as with the count data of interest here, there is no guarantee that this back transformation (${\tilde{Y}}_{t}=\Psi {\tilde{\beta }}_{t}$) will result in appropriate support for the elements of ${\tilde{Y}}_{t}$ (e.g., nonnegative values). This issue can be important in some applications, such as the analog forecasting problem of interest here, as we specify the $\beta_{t}$'s in a hierarchical model and require nonnegative values upon transformation back to physical space.

We employ nonnegative matrix factorization (NMF) (e.g., Lee & Seung, 2001) to enforce nonnegativity in the dimension reduction in the count data matrix. Given the $n_{y}\;\times \;T$ data matrix **Y**, NMF gives:

(1) Y≈ΨBΨ≥0,B≥0,

where Ψ is a $n_{y}\;\times \;n_{\beta }$ basis function matrix and the $n_{\beta }\;\times \;T$ matrix $B\equiv [\beta_{1},\ldots ,\;\beta_{T}]$ contains (random) projection coefficients. In reference to (1), the notation W≥0 for some matrix W, implies that each element of W is nonnegative. NMF has been applied in a variety of disciplines because of its ability to provide efficient dimension reduction while creating nonnegative basis functions. A number of different algorithms to conduct NMF have been proposed in the literature (e.g., Berry, Browne, Langville, Plemmons, & Paul Pauca, 2007), all with the goal of solving the following minimization problem:

(2) $$\min_{\Psi ,B\geq 0}\;D\left(Y,\;\Psi ,\;B\right)\;+\;R\left(\Psi ,\;B\right),$$

where D(Y,Ψ,B) is a loss function and R(Ψ,B) is some regularization function. Unfortunately, these NMF algorithms do not produce a unique factorization. Instead, they converge to a local minimum, thus producing different factorizations for different starting values (e.g., Boutsidis & Gallopoulos, 2008). To alleviate this nonuniqueness problem in our methodology, we use the Nonnegative Double Singular Value Decomposition (NNSVD) approach of Boutsidis and Gallopoulos (2008) to obtain starting values. Note that NNSVD was designed to produce fast convergence for sparse data structures (i.e., when **Y** contains a large number of zeros, as is the case with our BPS settling pattern data). The application to follow uses the so‐called off‐set NMF algorithm of Badea (2008).

### 2.4 Forcing vector dimension reduction

The purpose of the forcing variables, $\left\{x_{t^{\prime }}\right\}$, is to identify analogs to help predict the response variable. Further, the success of any analog forecasting model is largely determined by its ability to find robust analogs. If *n*_*x*_ is large, we typically must reduce the dimension of the process using spatial basis functions, $\Phi \equiv [\phi_{1},\ldots ,\;\phi_{n_{\alpha }}]$, where $\phi_{k}={\left(\phi_{k}\left(r_{1}\right),\ldots ,\;\phi_{k}\left(r_{n_{x}}\right)\right)}^{\prime }$. As with the response vector, if we assume linear projections, we can get projection coefficients by $\alpha_{t^{\prime }}={\left(\Phi^{\prime }\Phi \right)}^{-1}\Phi^{\prime }x_{t^{\prime }}$. McDermott and Wikle (2016) show that these projection coefficients can be combined to form time lagged *embedding matrices*. That is, let *q* represent the number of periods lagged back in time, then for period *t*, we can define the following $n_{\alpha }\;\times \;q$ embedding matrix:

(3) $$A_{t}=\left[\alpha_{t^{\prime }}\;,\;\;\alpha_{t^{\prime }-1},\ldots ,\;\alpha_{t^{\prime }-\left(q-1\right)}\right].$$

These embedding matrices are critical to the success of the analog forecasting model outlined below. For example, suppose we wanted to investigate whether the response variable at period *t*−1 was a robust analog for the response at period *t*. One could construct an embedding matrix $A_{t}$ corresponding to period *t* and another matrix $A_{t-1}$ for period t−1. We could assess the quality of $Y_{t-1}$ as an analog for the response at period *t*, by examining the “distance” between $A_{t}$ and $A_{t-1}$.

The selection of basis functions to obtain $\alpha_{t^{\prime }}$ can be flexible here and different choices of Φ could potentially produce different sets of analogs. For example, EOFs would be an obvious choice if linearity was assumed. However, there is scientific evidence of a nonlinear relationship between precipitation (which could potentially affect habitat conditions) and SST anomalies (e.g., Hoerling et al., 1997), so we investigated several nonlinear dimension reduction techniques for the waterfowl settling pattern application.

### 2.5 Hierarchical analog forecasting model

We now discuss the specifics of the spatiotemporal hierarchical Bayesian analog (HBA) forecasting model for count data. All of the stages of the presented HBA model are summarized in Table 1 below. As our responses $\left\{Y_{t}\;:\;t=1,\ldots ,\;T\right\}$ are count valued, we model the data with a Poisson distribution conditional on a spatiotemporal intensity process as:

(4) $$Y_{t}|\lambda_{t}∼\text{Poi}\left(\lambda_{t}\right),$$

**Table 1:** Hierarchical model summary

	Hierarchical Bayesian Analog Model
Data model:	$Y_{t}|\lambda_{t}∼\text{Poi}\left(\lambda_{t}\right)$
Process model:	$\beta_{t}|B_{-t},\;\tilde{\Theta }∼{\text{TN}}_{\left[0,\infty \right)}\left(\max\left\{h\left(B_{-t}\omega_{t},\;\sigma_{\eta }^{2}\right),\;\epsilon \right\}\;\sigma_{\eta }^{2}I\right)$
where	$B_{-t}\equiv \left[\beta_{1},\ldots ,\;\beta_{t-1},\;\beta_{t+1},\;\ldots ,\;\;\beta_{T}\right]$
	$\omega_{t}\equiv {\left(\omega \left(A_{t},\;\;A_{1},\;\;\theta \right),\ldots ,\;\;\omega \left(A_{t},\;\;A_{t-1},\;\;\theta \right),\;\;\omega \left(A_{t},\;\;A_{t+1},\;\;\theta \right),\ldots ,\;\;\omega \left(A_{t},\;\;A_{T},\;\theta \right)\right)}^{\prime }$
Parameter model:	$q∼\text{DU}(q_{\min},\;q_{\max})$ $m∼\text{DU}(m_{\min},\;m_{\max})$
	$\theta_{1}∼\text{IG}\left(a_{1},\;\;b_{1}\right)$ $\sigma_{\eta }^{2}∼\text{IG}\left(a_{2},\;\;b_{2}\right)$
Hyperparameters:	ϵ, $q_{\min}$, $q_{\max}$, $m_{\min}$, $m_{\max}$, $a_{1}$, $b_{1}$, $a_{2}$, $b_{2}$, $\theta_{2}$

where $\left\{\lambda_{t}\;:\;t=1,\ldots ,\;T\right\}$ is the $n_{y}$‐dimensional intensity process at locations $\left\{s_{1},\ldots ,\;s_{n_{y}}\right\}$. Using the basis functions from the NMF approximation (1), let $\lambda_{t}\;=\;\Psi \beta_{t}$. Recall, the NMF guarantees Ψ≥0 and thus, for $\lambda_{t}$ to be nonnegative, the distribution for $\beta_{t}$ should have nonnegative support. If we denote the model parameters by $\tilde{\Theta }$ (see below), then for period *t*, the process model on $\beta_{t}$ is given by the truncated normal distribution:

(5) $$\beta_{t}|B_{-t},\;\tilde{\Theta }∼{\text{TN}}_{\left[0,\infty \right)}\left(\text{max}\left\{h\left(B_{-t}\omega_{t},\;\sigma_{\eta }^{2}\right),\;\epsilon \right\},\;\sigma_{\eta }^{2}I\right),$$

where, for period *t*, we define $B_{-t}\;\equiv \;\left[\beta_{1},\ldots ,\;\beta_{t-1},\beta_{t+1},\ldots ,\;\beta_{T}\right]$ as the matrix of possible analogs and $\omega_{t}=(\omega \left(A_{t},\;A_{1},\;\theta \right),\ldots ,\;\omega \left(A_{t},\;A_{t-1},\;\theta \right),\;$ $\omega \left(A_{t},\;A_{t+1},\;\theta \right),\ldots ,\;\omega \left(A_{t},\;A_{T},\;\theta \right))\prime$, as the weight associated with each of the potential analogs. Thus, a weighted prediction of the new $\beta_{t}$ is based on the linear combination of past $\beta_{t}$ values, $B_{-t}\omega_{t}$. Due to the form of (5), in particular the weighted averaged (i.e., $B_{-t}\omega_{t}$), we found that using a log‐Gaussian formulation in (5) failed to preserve the correct scale of the analogs.

Further, as described in Cangelosi and Hooten (2009), for a normal density left‐truncated at zero, the mean is biased and this bias increases for values close to zero (which is the case for many elements of $\beta_{t}$) as the left tail of the distribution has been distorted from the truncation at zero. In equation (5), h(·) is the bias correction function presented in Cangelosi and Hooten (2009). The need for the constant ϵ arises since as $B_{-t}\;\omega_{t}\;\to \;0$, we have $h(B_{-t}\;\;\omega_{t},\;\sigma_{\eta }^{2})\to -\infty$. Thus, ϵ is set to an arbitrarily small value for computational purposes. The weights ($\omega_{t}$) in (5) are critical to the success of the analog forecasting model presented here. For example, during the training of the model, these weights determine how much each potential analog in $B_{-t}$ is weighted in order to predict $\beta_{t}$. We describe our choice of weights in the next section. It is important to note that, although the weights are applied to the potential analogs in a linear fashion (i.e., $B_{-t}\;\omega_{t}$), the resulting prediction for $\beta_{t}$ can be considered nonlinear as the weights are determined by a nonlinear function (i.e., the Gaussian kernel).

**Figure 1:**
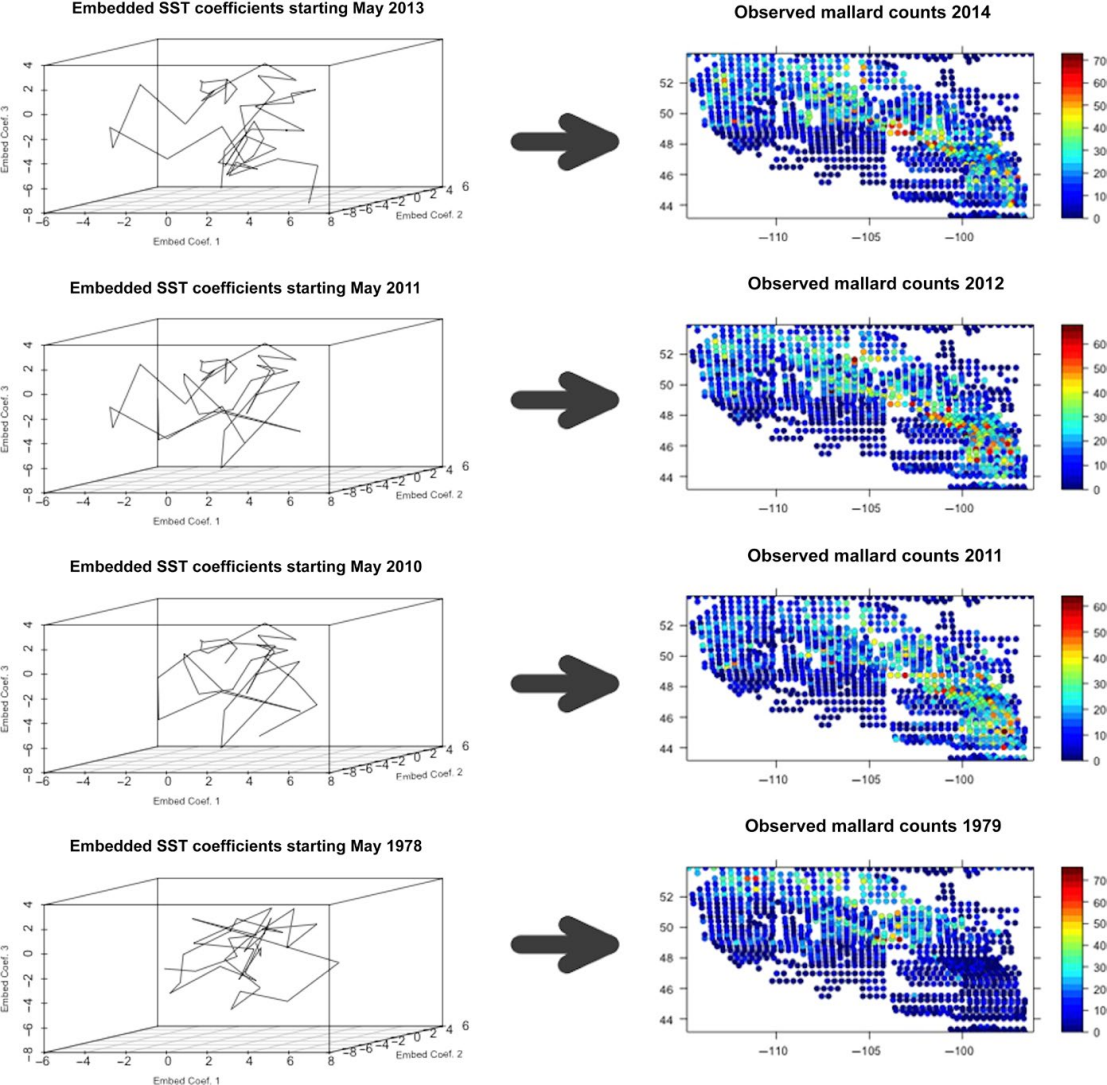
Example illustrating analog forecasting of waterfowl counts for 2014. Attractor manifold plots on the left are examples of embedding matrices (see (3)), where $n_{\alpha }=3$ and *q* = 50 (months). The three plots below the plot starting in May 2013 (left column) are examples of nearest neighbor analogs. These three neighbors are selected based on their similarity in shape (Procrustes distance) to the attractor time series for May 2013 (i.e., the initial condition for a one‐year‐ahead forecast for May 2014). Each of the three nearest neighbors is associated with a corresponding waterfowl pattern (right column). The three waterfowl patterns for the nearest neighbors are each then appropriately weighted to form a forecast for 2014

The choice of analog weights and the analog “neighborhood’’ is closely connected and important considerations in analog forecasting. Let $N_{m}\left(A_{t}\right)$ denote the neighborhood of the analog $A_{t}$ for period *t*, where the number of nearest neighbors considered is represented by *m*. Defining d(·) as a distance metric and $\theta =\{\theta_{1},\theta_{2}\}$ as a set of kernel‐dependent parameters, we have the following kernel weight function:

(6) $$\tilde{\omega }\left(A_{t},\;A_{\ell },\;\theta \right)=\left\{\begin{array}{cc} \text{exp}\left(\frac{-d{\left(A_{t},A_{\ell };\theta_{2}\right)}^{2}}{2\theta_{1}}\right), & \text{if}\;\;\;\;A_{\ell }\in N_{m}\left(A_{t}\right)\\ 0, & \text{if}\;\;\;\;A_{\ell }\notin N_{m}\left(A_{t}\right), \end{array}\right.$$

for ℓ≠t, where $\theta_{1}$ is a kernel smoothing parameter and $\theta_{2}$ is a parameter associated with the distance function (see the Appendix). Let, $\omega (A_{t},\;\;A_{\ell },\;\;\theta )$ be the normalized version of $\tilde{\omega }\left(A_{t},\;\;A_{\ell },\;\;\theta \right)$, where the normalization is accomplished by dividing by the sum of $\tilde{\omega }\left(A_{t},\;\;A_{\ell },\;\;\theta \right)$ across all T−1 potential analogs for period *t*. Any valid distance metric d(·) can be applied here; for example, analog forecasting methods traditionally use Euclidean distance. However, analog forecasting relies on identifying analogs that not only resemble the current state of the system but also move forward in a similar manner. For this reason, analogs that share a similar trajectory in phase space as the current trajectory of the system will produce the most successful forecasts. Procrustes distance (e.g., see Hastie, Tibshirani, & Friedman, 2013) is a multivariate distance metric that transforms a comparison object (i.e., $A_{\ell }$) to a target object (i.e., $A_{t}$), before calculating the Frobenius matrix norm between the target object and the transformed comparison object. Therefore, by defining $d(A_{t},\;A_{\ell };\;\theta_{2})$ as the Procrustes distance (see the Appendix for the full details, including the specification of $\theta_{2}$), we are able to compare the shape, and thus, the trajectory, between two embedding matrices (see Figure 1 for a visual example). In the definition of $A_{t}$, we let *q* represent the number of lagged time periods when forming $A_{t}$. As different values of *q* will lead to different embedding matrices, and thus potentially different analogs, we estimate *q* and give it a discrete uniform prior such that, $q∼\text{DU}(q_{\min},\;q_{\max})$. We also assign a discrete uniform prior to the number of neighbors parameter, $m∼\text{DU}(m_{\min},\;m_{\max})$. Finally, $\theta_{1}$ and $\sigma_{\eta }^{2}$ are both assigned inverse‐gamma priors, $\theta_{1}∼\text{IG}\left(a_{1},\;b_{1}\right)$ and $\sigma_{\eta }^{2}∼\text{IG}\left(a_{2},\;b_{2}\right)$.

Sampling from the posterior distribution is accomplished with Markov chain Monte Carlo (MCMC) methods (e.g., Robert & Casella, 2004). Due to the lack of conjugacy, all parameters are updated with a Metropolis—Hastings step (see the outline in the Appendix). During each iteration of the MCMC sampler, parameters are sampled using data from training periods, t=1,…,T. At this stage, all prediction is “in‐sample.” For period T+1, out‐of‐sample forecasts are then drawn from the posterior prediction distribution, $Y_{T+1}^{\left(\ell \right)}\;∼\;\text{Poi}\left(\Psi \beta_{T+1}^{\left(\ell \right)}\right)$, after each iteration, ℓ, of the sampler. By defining, $B_{T+1}^{\left(\ell \right)}=\left[\beta_{1}^{\left(\ell \right)},\;\ldots ,\;\;\beta_{T}^{\left(\ell \right)}\right]$ and $\omega_{T+1}^{\left(\ell \right)}={\left(\omega \left(A_{T+1},\;\;A_{1},\;\;\theta^{\left(\ell \right)}\right),\ldots ,\;\omega \left(A_{T+1},\;\;A_{T},\;\;\theta^{\left(\ell \right)}\right)\right)}^{\prime }$, the projection coefficients for period T+1 can be forecasted for the $\ell^{\mathrm{th}}$ iteration as, $\beta_{T+1}^{\left(\ell \right)}=B_{T+1}^{\left(\ell \right)}\omega_{T+1}^{\left(\ell \right)}$. In this example, $A_{T+1}$ is the initial condition for which we seek matching past analogs. Then, from the definition of (3), the first element of $A_{T+1}$ is $\alpha_{T^{\prime }+1}$, which is lagged τ periods behind the forecast target time, T+1, thus leading to a τ‐period ahead forecast of $Y_{T+1}$ (see Figure 1 for an illustrative example).

### 2.6 Model setup

We evaluate the predictive ability of the model by considering forecasts of waterfowl counts in 2009 and 2014, while also producing hindcasts for 1999. The year 2009 was chosen due to the relative lack of correlation between the mallard counts in 2009 and the prior year. Further, we choose to consider 1999 because it was a strong La Niña year, which allows us to demonstrate how the model can effectively forecast years where waterfowl patterns may change due to alternating habitat conditions. All of the data prior to the respective year is used for training 2009 and 2014, while the hindcast is implemented by training on all of the data except the counts for 1999. We make one‐year‐ahead forecasts for all time periods by setting τ=12.

We compare the forecasting skill of the HBA model with a fairly state‐of‐the art hierarchical Bayesian Poisson space–time model (referred to as the PST model). The PST model is comprised of a Poisson data model, $Y_{t}|\lambda_{t}∼\text{Poi}\left(\lambda_{t}\right)$, and process model defined as, $\text{log}\left(\lambda_{t}\right)∼\text{Gau}\left(\mu +\Psi \alpha_{t},\;\sigma_{\epsilon }^{2}I\right)$. Here, μ is a spatially indexed mean (modeled with spatial covariates), and $\alpha_{t}$ are projection coefficients formed from kernel principal component analysis. The projection coefficients are modeled with a reduced rank vector‐autoregressive (VAR) structure such that, $\alpha_{t}∼\text{Gau}\left(H\alpha_{t-1},\;\Sigma_{\gamma }\right)$ (e.g., see Chapter 7 of Cressie & Wikle, 2011). Specification of the process model for the PST model can be thought of as a linear version of the regime‐dependent nonlinear model presented in Wu et al. (2013). Comparison of the posterior predictions for the HBA and PST model is carried out using mean squared prediction error (MSPE) and the correlation between the forecasted and observed values as in McDermott and Wikle (2016).

The HBA model was implemented for all forecasted years with the same tuning parameters and prior distributions. Note, as $n_{\beta }$ increases, the NMF basis function approximation in (1) generally becomes more accurate. Because there is a computational cost to using higher values of $n_{\beta }$, we found that $n_{\beta }=14$ appropriately balanced computational efficiency with the accuracy of the approximation.

Regarding the SST basis functions, in addition to the more traditional empirical orthogonal function (EOFs; i.e., spatiotemporal principal components) linear dimension reduction, we implemented the following nonlinear dimension reduction methods: local linear embeddings (e.g., Roweis & Saul, 2000), diffusion maps (e.g., Coifman & Lafon, 2006), kernel principal component analysis (KPCA) (e.g., Scholkopf, Smola, & Muller, 1998), and Laplacian eigenmaps (e.g., Belkin & Niyogi, 2001). Our analysis found Laplacian eigenmaps to be the most helpful of these nonlinear methods for identifying robust analogs. Therefore separate models, one with EOF basis functions (HBA1) and a second model with Laplacian eigenmap basis functions (HBA2), were implemented. Approximately 82% of the variation in the SST data was accounted for by the first 16 EOFs (i.e., $n_{\alpha }=16$). Laplacian eigenmaps are calculated through an eigenvector decomposition of a Laplacian matrix, whose construction involves an adjacency matrix formed through either a kernel or a nearest neighbor approach (e.g., Belkin & Niyogi, 2001). We implemented the nearest neighbor approach, with $n_{\alpha }=16$ again, by sampling the number of neighbors as a parameter in the MCMC sampler over the following grid: {6+3×d:d=0,…10}. Although we are applying Takens’ embedding theorem (Takens, 1981) with a relatively short temporal span (i.e., approximately 40 years) using 16 state variables to represent the SST data, we seem to be able to counterbalance the effects of a shorter temporal span. A further examination of this trade‐off between the temporal span and number of state variables is beyond the scope of this study, but should be investigated elsewhere. This span is short enough that potential nonstationarities in the SST data are not a major concern.

We used a value of 10^−6^ for the ϵ parameter in (5); the model did not seem overly sensitive to this choice. For *q* and *m*, we assigned priors q∼DU(30,60) and m∼DU(1,15), respectively. The kernel and process error parameters are given inverse‐gamma priors: $\theta_{1}∼\text{IG}\left(2.02,\;0.102\right)$ (which is only moderately informative in comparison with the small scale of the Gaussian kernel in (6)) and $\sigma_{\eta }^{2}∼\text{IG}\left(0.001,\;0.001\right)$. All models were run for 20,000 iterations with the first 2,000 considered burn‐in.

## 3 RESULTS

Prediction skill of the HBA and PST models was evaluated through calculation of the MSPE, defined as the mean of the squared differences between the posterior predicted means and the observed counts averaged across all spatial locations. The correlation between the observed counts and the mean of the posterior predictions was also used to evaluate the forecasting models, as is often considered for spatiotemporal prediction (e.g., Wilks, 2006). As displayed in Table 2, the HBA model outperformed the PST model in both 2009 and 2014, in that the HBA models had higher correlations and lower MSPE values for the two forecasted years. For 1999 and 2009, the EOF‐based analog model (HBA1) produced the most accurate results and the Laplacian eigenmaps model (HBA2) outperformed the EOF model in 2014. The correlation and MSPE for the hindcast appear to align with the results for the two forecasted years. We applied the model to several other holdout years (not shown here) and found similar results, with the HBA always performing as well or better than the PST model and with the HBA1 generally, but not always, outperforming HBA2. To examine the model performance with a shorter time period, we also ran HBA1 for 2009 over a 15‐year period (i.e., we only trained the model on data from the 15 years prior to 2009). With fewer potential analogs, the model performed slightly worse over the shorter temporal span (i.e., the MSPE was 69.941 and the correlation was 66.176%).

**Table 2:** Results based on the posterior predictive distribution for the two HBA models, and the PST model. Models are compared via mean squared prediction error (MSPE) and correlation (Corr) of the forecasted values with observed values. The two HBA models are implemented across 3 holdout years, while the PST model is only evaluated for 2009 and 2014

Model	1999		2009		2014	
	MSPE	Corr	MSPE	Corr	MSPE	Corr
HBA1	58.822	83.031%	63.056	70.307%	59.694	78.699%
HBA2	62.575	82.856%	70.085	66.808%	57.799	79.446%
PST	–	–	73.435	66.103%	69.975	77.780%

**Figure 2:**
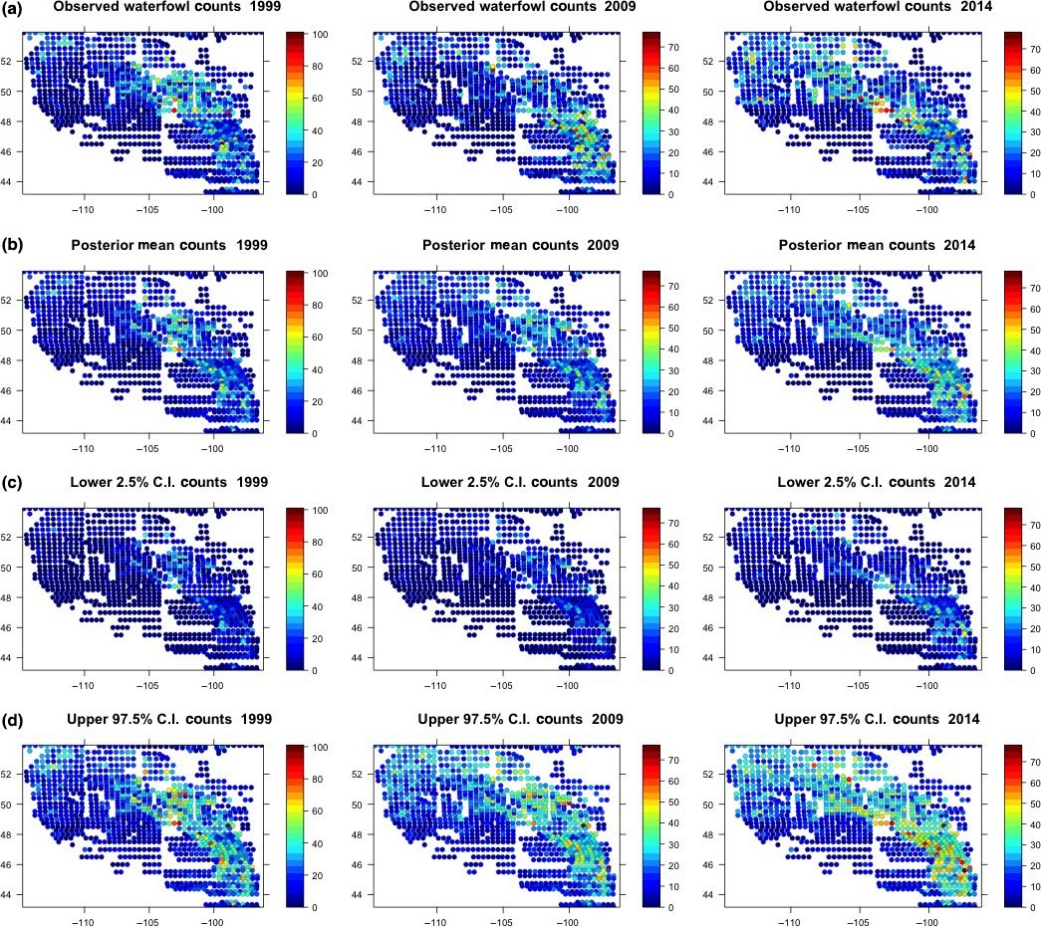
Summary of the posterior predictive results for the HBA1 model. (a) Observed waterfowl counts for 1999, 2009, and 2014 (left to right), (b) means of the posterior predictive distribution for each year, (c) lower 2.5th percentile from the posterior predictive distribution, and (d) upper 2.5th percentile form the posterior predictive distribution for each year

The hindcast and prediction maps (observed, forecasted mean, site‐specific lower 2.5th, and upper 2.5th percentiles) demonstrate that the pattern of forecasted counts captures the overall pattern of the observed counts (Figure 2). Close examination of the uncertainty maps show that a majority of the observed counts fall within the displayed 95% credible intervals. The 1999 hindcast correctly predicts a pattern of mallards settling more heavily in the northern region of the domain, a year when the mallard population was estimated to be 10.8 million, which was the second largest population size estimated for the species since 1955 (U.S. Fish and Wildlife Service, 1999). There is often substantial variation in settling patterns of waterfowl which are typically tied to climate conditions and the 2009 and 2014 waterfowl counts demonstrate that variability. In both 2009 and 2014, waterfowl settled in greater densities in the southeast region of the area with some of the highest densities of waterfowl settling in North and South Dakota. In 2009, above‐average moisture was observed in these areas with a 62% increase in mallard numbers in eastern North and South Dakota compared with 2008 (U.S. Fish and Wildlife Service, 2009). Similarly, above‐average precipitation was observed in 2014, but estimated mallard numbers were reduced by 28% in North and South Dakota when compared with 2013 (U.S. Fish and Wildlife Service, 2014). Thus, there can be substantial changes in population estimates and settling patterns, yet our predictions demonstrate the ability of our model to accurately reflect settling patterns for mallards during these time periods.

## 4 DISCUSSION

Overall, many of the aspects of analog forecasting that originally made it appealing to ecologists are retained by the HBA model. The model has few parameters and performs well with data from a relatively short temporal span. Unlike other analog forecasting methods, the HBA allows users to properly quantify uncertainty in a rigorous framework. With the growing number of high‐dimensional spatial–temporal ecological datasets, analog forecasting in a hierarchical framework can provide ecologists with a rich framework for making accurate forecasts with principled uncertainty quantification. The count‐based spatiotemporal hierarchical Bayesian analog model methodology developed here was successful in that it produced forecasts that had high correlations with observed counts, along with outperforming a hierarchical Bayesian Poisson space–time model (in terms of MSE and correlation).

The result that waterfowl settled more consistently in the northern half of the region of interest in 1999 despite the lack of correlation with patterns from the previous year was promising. We suspect that poor habitat conditions due to drought (e.g., Wu et al., 2013), possibly linked to the tropical Pacific La Niña anomaly (e.g, Philander, 1990; Hoerling et al., 1997), could help explain why many waterfowl overflew the southern region in 1999 (e.g, Hansen & McKnight, 1964; Sorenson et al., 1998). The distribution of migratory birds is notoriously affected by climatic factors. For example, the timing of waterfowl migrations might be affected by climate as can the use of stopover sites, the distance travelled, and the ultimate location for settling (Schummer, Cohen, Kaminski, Brown, & Wax, 2014). In fact, several studies describe how migratory birds, including waterfowl, adjust their migration strategy depending on various weather conditions (McEvoy, Roshier, Ribot, & Bennett, 2015). In the presence of climate change, the ability to effectively model migratory bird migration patterns becomes even more important to wildlife managers. For waterfowl, modeling how traditional waterfowl migratory routes might change, and the fidelity of waterfowl to specific routes, becomes paramount to effective management because those routes are used to delineate harvest management boundaries. Our model has demonstrated the capacity to be responsive to such changes, and to our knowledge, it is the only existing spatiotemporal nonlinear analog model that quantifies forecast uncertainty.

These models provide wildlife managers accurate forecasts and informative intervals which would allow them to make more informed harvest management decisions, better understand the reasons for the settling patterns observed, and assess how waterfowl are responding to habitat treatments. For example, a huge emphasis of some federal government and nongovernmental organizations has involved the conservation and restoration of critical wetland habitats for the benefit of waterfowl. During their 80 years of existence, Ducks Unlimited has improved 13 million acres of wetland habitat. Through application of our modeling results, managers could assess whether waterfowl respond favorably to habitat manipulations by settling in those environments. Also, these results can help highlight regions and locales that should be prioritized based on duck settlement patterns (i.e., waterfowl hotspots). In that way, managers can be strategic and effective in prioritizing their habitat management plans (e.g., Bonnot, Thompson, Millspaugh, & Jones‐Farrand, 2013). In this context, the concept of “Strategic Habitat Conservation” has been promoted by the U.S. Fish and Wildlife Service as a means of integrating research, management objectives, monitoring, and habitat design strategies for making decisions about habitat (US Fish and Wildlife Service and others, 2006). Our work provides proof of concept for application of hierarchical spatiotemporal forecasting models to aid in prioritizing habitat management decisions based on waterfowl settling patterns.

Due to the preponderance of zeros present in the waterfowl data, the assumption of equidispersion implicit in the data model (i.e., equation (4)) is likely violated. Wu et al. (2013) attempted to deal with the potential underdispersion in such data using a Conway‐Maxwell Poisson data model. Here, we deal with this potential problem using NMF to reduce the overall underdispersion. However, a more rigorous way to deal with the underdispersion is to use a zero‐inflated Poisson (ZIP) data model (e.g., Ver Hoef & Jansen, 2007; Wikle & Anderson, 2003). Importantly, any of the various methods throughout the literature that account for underdispersion could be integrated into the presented model by adjusting the data model.

By placing analog forecasting within a hierarchical Bayesian paradigm, there are a multitude of ways in which the methodology could be extended. It should be noted that differences in the forecasts between the HBA1 and HBA2 model can be attributed to a difference in the selection of analogs. This suggests that allowing the model to simultaneously consider multiple types of basis functions is an obvious extension of the model. Through the use of a mixture model, one could potentially jointly model two or more types of basis functions. Such an approach may be useful for forecasting seasonal or yearly settling patterns that are influenced both linearly and nonlinearly by some high‐dimensional variable.

Count data in ecology are ubiquitous and the model we developed is an ideal alternative to currently available quantitative methods. Ecologists routinely collect count data through visual surveys, such as the waterfowl dataset used herein, or through use of other remote technologies. For example, rapid advancement of radio‐tracking technology (e.g, Kays, Crofoot, Jetz, & Wikelski, 2015) and remote‐sensed cameras (e.g, He et al., 2016) has transformed the way ecologists collect count data. These widely used technologies have also changed the type of data obtained both in terms of amount and structure of resulting data. In particular, these technologies result in large data structures with spatial and temporal dependencies and our model provides an appropriate way to address these complexities while quantifying uncertainty in a rigorous manner. Often, these count data are used by ecologists to assess settling patterns, habitat relationships, or impacts of weather conditions and predict future states. For example, migration routes of terrestrial mammals are imperiled (e.g, Berger & Cain, 2014) and there is much effort to identify and predict use of important migration corridors. However, timing and use of migration corridors are affected by weather and other factors such as human disturbance. Our model provides an alternative to model and project use of these important areas while revealing factors affecting their use. Indeed, although analog methods typically require quite a bit of data to develop the analog libraries, it does not necessarily require historical data over long time spans. So, processes operating on faster time scales, for which relatively high‐frequency observations are available, can be considered within this framework and used to develop predictions. Such results would have important policy decisions in wildlife management. Thus, we envision numerous applications of this model and its extensions.

## CONFLICT OF INTEREST

None.

## DATA ACCESSIBILITY

The raw indicated pair counts (for the waterfowl settling pattern data) are publicly available through the FWS Division of Migratory Management (https://migbirdapps.fws.gov/). The monthly sea surface temperature (SST) data are publicly available from the National Oceanic and Atmospheric Administration (NOAA) Extended Reconstruction Sea Surface Temperature (ERSST) data (http://www.esrl.noaa.gov/psd/).

## AUTHOR CONTRIBUTION

Patrick McDermott wrote the initial manuscript. Christopher Wikle and Joshua Millspaugh provided many edits for multiple versions of the manuscript. The model was implemented by Patrick McDermott, with guidance from Christopher Wikle. Joshua Millspaugh provided ecological guidance and context throughout the writing and editing process.
