# Supplementary material for: A hierarchical spatiotemporal analog forecasting model for count data
Source: Ecol Evol. 2017 Dec 7;8(1):790–800. doi: 10.1002/ece3.3621 (PMC5756884; doi:10.1002/ece3.3621)
Supplement: Supplementary file 1 [file ECE3-8-790-s001.pdf]

Appendix: A Hierarchical Spatio-Temporal Analog Forecasting Model for Count Data

Appendix A: Markov chain Monte Carlo Algorithm

The following details the MCMC algorithm used to implement the HBA model outlined above. Let $\ell = 1, \ldots, L$ represent the current iteration.

1. Sample $\beta_{j,t}^{(\ell)}$ using componentwise Metropolis-Hastings updates.

   Let $\widetilde{\boldsymbol{\Theta}}^{(\ell-1)} = \{m^{(\ell-1)}, q^{(\ell-1)}, \theta_1^{(\ell-1)}, \sigma_\eta^{2(\ell-1)}\}$. Generate a proposal value from $\log(\beta_{j,t}^*) \sim$ Gau$(\log(\beta_{j,t}^{(\ell-1)}), \zeta_{j,t})$ (where $\zeta_{j,t}$ is a tuning parameter) and calculate the following vectors:
   $\boldsymbol{\beta}_t^0 = (\beta_{1,t}^{(\ell)}, \ldots, \beta_{j-1,t}^{(\ell)}, \beta_{j,t}^{(\ell-1)}, \beta_{j+1,t}^{(\ell-1)}, \ldots, \beta_{n_\beta,t}^{(\ell-1)})'$
   $\boldsymbol{\beta}_t^* = (\beta_{1,t}^{(\ell)}, \ldots, \beta_{j-1,t}^{(\ell)}, \beta_{j,t}^*, \beta_{j+1,t}^{(\ell-1)}, \ldots, \beta_{n_\beta,t}^{(\ell-1)})'$
   $\boldsymbol{\beta}_j^0 = (\beta_{j,1}^{(\ell)}, \ldots, \beta_{j,t-1}^{(\ell)}, \beta_{j,t}^{(\ell-1)}, \beta_{j,t+1}^{(\ell-1)}, \ldots, \beta_{j,T}^{(\ell-1)})'$
   $\boldsymbol{\beta}_j^* = (\beta_{j,1}^{(\ell)}, \ldots, \beta_{j,t-1}^{(\ell)}, \beta_{j,t}^*, \beta_{j,t+1}^{(\ell-1)}, \ldots, \beta_{j,T}^{(\ell-1)})'$.

   Next, calculate the following Metropolis-Hastings ratio:

$$R(\beta_{j,t}^*, \beta_{j,t}^{(\ell-1)}) = \frac{\left[\boldsymbol{Y}_t | \boldsymbol{\beta}_t^*, \boldsymbol{\Psi}\right] \prod_{t=1}^{T} \left[\boldsymbol{\beta}_{j,t}^* | \boldsymbol{\beta}_{j,-t}^*, \widetilde{\boldsymbol{\Theta}}^{(\ell-1)}\right]}{\left[\boldsymbol{Y}_t | \boldsymbol{\beta}_t^0, \boldsymbol{\Psi}\right] \prod_{t=1}^{T} \left[\boldsymbol{\beta}_{j,t}^0 | \boldsymbol{\beta}_{j,-t}^0, \widetilde{\boldsymbol{\Theta}}^{(\ell-1)}\right]}.$$

Set $\beta_{j,t}^{(\ell)} = \beta_{j,t}^{*}$ with probability $\min\{1, R(\beta_{j,t}^{*}, \beta_{j,t}^{(\ell-1)})\}$; otherwise $\beta_{j,t}^{(\ell)} = \beta_{j,t}^{(\ell-1)}$. Repeat for $j = 1, \ldots, n_\beta$ and $t = 1, \ldots, T$. This derivation assumes each $\beta_{j,t}$ are sampled in the same order each iteration, in practice the order could be random and change each iteration.

2. Sample m by performing inverse transform sampling with the discrete grid, $\{m_i^* : i = 1, \ldots, (m_{max} - m_{min}, +1)\}$. Evaluate the following probability:

$$p_i = \prod_{t=1}^{T} \left[\boldsymbol{\beta}_t^{(\ell)} \mid \boldsymbol{\beta}_{-t}^{(\ell)}, m_i^*, q^{(\ell-1)}, \theta_1^{(\ell-1)}, \sigma_\eta^{2(\ell-1)}\right],$$

for each m_i^*. Calculate the C.D.F. by normalizing each p_i (i.e., $\tilde{p}_i = \frac{p_i}{\sum_i p_i}$) and use inverse transform sampling to sample $m^{(\ell)}$.

3. Sample q by performing inverse transform sampling with the discrete grid, $\{q_k^* : k = 1, \ldots, (q_{max} - q_{min}, +1)\}$. Evaluate the following probability:

$$p_k = \prod_{t=1}^{T} \left[\boldsymbol{\beta}_t^{(\ell)} \mid \boldsymbol{\beta}_{-t}^{(\ell)}, m^{(\ell)}, q_k^*, \theta_1^{(\ell-1)}, \sigma_\eta^{2(\ell-1)}\right],$$

for each q_k^*. Calculate the C.D.F. by normalizing each p_k and use inverse transform sampling to sample $q^{(\ell)}$.

4. Sample $\theta_1^{(\ell)}$ with a Metropolis-Hastings step. Generate a proposal value from $\log(\theta_1^*) \sim \text{Gau}(\log(\theta_1^{(\ell-1)}), \sigma_{\theta_1}^2)$ and calculate the following Metropolis-Hastings ratio:

$$R(\theta_1^*, \theta_1^{(\ell-1)}) = \frac{\prod_{t=1}^{T} \left[\boldsymbol{\beta}_t^{(\ell)} | \boldsymbol{\beta}_{-t}^{(\ell)}, m^{(\ell)}, q_k^{(\ell)}, \theta_1^*, \sigma_\eta^{2(\ell-1)}\right] \left[\theta_1^* | a_1, b_1\right]}{\prod_{t=1}^{T} \left[\boldsymbol{\beta}_t^{(\ell)} | \boldsymbol{\beta}_{-t}^{(\ell)}, m^{(\ell)}, q_k^{(\ell)}, \theta_1^{(\ell-1)}, \sigma_\eta^{2(\ell-1)}\right] \left[\theta_1^{(\ell-1)} | a_1, b_1\right]}.$$

Set $\theta_1^{(\ell)} = \theta_1^*$ with probability $\min\{1, R(\theta_1^*, \theta_1^{(\ell-1)})\}$; otherwise $\theta_1^{(\ell)} = \theta_1^{(\ell-1)}$.

5. Sample $\sigma_\eta^{2(\ell)}$ with a Metropolis-Hastings step. Generate a proposal value from $\log(\sigma_\eta^{2*}) \sim \text{Gau}(\log(\sigma_\eta^{2(\ell-1)}), \sigma^2_{\sigma^2_\eta})$ and calculate the following Metropolis-Hastings ratio:

$$R(\sigma_\eta^{2*}, \sigma_\eta^{2(\ell-1)}) = \frac{\prod_{t=1}^{T} \left[\boldsymbol{\beta}_t^{(\ell)} | \boldsymbol{\beta}_{-t}^{(\ell)}, m^{(\ell)}, q_k^{(\ell)}, \theta_1^{(\ell)}, \sigma_\eta^{2*}\right] \left[\sigma_\eta^{2*} | a_2, b_2\right]}{\prod_{t=1}^{T} \left[\boldsymbol{\beta}_t^{(\ell)} | \boldsymbol{\beta}_{-t}^{(\ell)}, m^{(\ell)}, q_k^{(\ell)}, \theta_1^{(\ell)}, \sigma_\eta^{2(\ell-1)}\right] \left[\sigma_\eta^{2(\ell-1)} | a_2, b_2\right]}.$$

Set $\sigma_\eta^{2(\ell)} = \sigma_\eta^{2*}$ with probability $\min\{1, R(\sigma_\eta^{2*}, \sigma_\eta^{2(\ell-1)})\}$; otherwise $\sigma_\eta^{2(\ell)} = \sigma_\eta^{2(\ell-1)}$.

Appendix B: Procrustes Distance

Suppose we have a target object matrix $\mathbf{E}$ and a comparison object matrix $\mathbf{F}$. It is assumed that $\mathbf{E}$ and $\mathbf{F}$ have the same dimension. To compare the two objects the comparison object is superimposed onto the target object through scaling, rotation, and translation. This transformation is carried out by using the scaling parameter θ_2 and the rotation matrix $\mathbf{R}$. The Procrustes distance between $\mathbf{E}$ and $\mathbf{F}$ is defined as:

$$d(\mathbf{E}, \mathbf{F}; \theta_2) = ||\mathbf{E} - \theta_2 \mathbf{F}\mathbf{R}||_F \ ,$$

where the "F" subscript denotes the Frobenius matrix norm. To calculate $\mathbf{R}$ we need to center $\mathbf{E}$ and $\mathbf{F}$ by their respective column means to create $\widetilde{\mathbf{E}}$ and $\widetilde{\mathbf{F}}$. Next, we calculate the singular value decomposition of $\widetilde{\mathbf{E}}\widetilde{\mathbf{F}}' = \mathbf{U}\mathbf{D}\mathbf{V}'$ and let $\mathbf{R} = \mathbf{U}\mathbf{V}'$. The positive scaling parameter is set such that $\theta_2 = tr(\mathbf{D})/||\mathbf{F}||_F^2$.
